# Synthesis of Cryolite (Na_3_AlF_6_) from Secondary Aluminum Dross Generated in the Aluminum Recycling Process

**DOI:** 10.3390/ma13173871

**Published:** 2020-09-02

**Authors:** Bingbing Wan, Wenfang Li, Wanting Sun, Fangfang Liu, Bin Chen, Shiyao Xu, Weiping Chen, Aihua Yi

**Affiliations:** 1 School of Materials Science and Engineering, Dongguan University of Technology, Dongguan 523000, China; wanbb070320@mail.xjtu.edu.cn (B.W.); sunwt628@mail.xjtu.edu.cn (W.S.); chenbin@dgut.edu.cn (B.C.); yiah@dgut.edu.cn (A.Y.); 2 School of Materials Science and Engineering, Xi’an Jiaotong University, Xi’an 710049, China; 3 Guangdong Key Laboratory for Advanced Metallic Materials Processing, South China University of Technology, Guangzhou 510640, China; 2018309@dgut.edu.cn; 4 Department of Electromechanical Engineering, Guangdong University of Science and Technology, Dongguan 523083, China; liufangfang@gdust.edu.cn

**Keywords:** secondary aluminum dross, recycling, extraction, synthetic cryolite

## Abstract

Secondary aluminum dross (SAD) is regarded as a solid waste of aluminum recycling process that creates serious environmental and health concerns. However, SAD can also be used as a good source of aluminum, so that utilizing the SAD for the production of valuable products is a promising approach of recycling such waste. In the present work, a novel eco-friendly three-step process was proposed for the synthesis of cryolite (Na_3_AlF_6_) from the SAD, and it consisted of (1) water-washing pretreatment of SAD, (2) extraction of Al component via pyro-hydrometallurgy, including low-temperature alkaline smelting, water leaching and purification of leachate in sequence, (3) precipitation of cryolite from the purified NaAlO_2_ solution using the carbonation method. By analysis of the parameter optimization for each procedure, it was found that the maximum hydrolysis efficiency of aluminum nitride (AlN) in the SAD was around 68.3% accompanied with an extraction efficiency of Al reaching 91.5%. On this basis, the cryolite of high quality was synthesized under the following optimal carbonation conditions: reaction temperature of 75 °C, NaAlO_2_ concentration of 0.11 mol/L, F/(6Al) molar ratio of 1.10, and 99.99% CO_2_ gas pressure, and flow rate of 0.2 MPa and 0.5 L/min respectively. The formation of Na_3_AlF_6_ phase can be detected by XRD. The morphological feature observed by SEM revealed that the as-synthesized cryolite had a polyhedral shape (~1 μm size) with obvious agglomeration. The chemical composition and ignition loss of the as-synthesized cryolite complied well with the requirements of the Chinese national standard (GB/T 4291-2017).

## 1. Introduction

With the extensive development of secondary aluminum industry, secondary aluminum dross (SAD) is widely generated during aluminum recycling process through remelting with salt flux. In general, the SAD primarily contains Al_2_O_3_, aluminum metal, AlN, NaCl, KCl, SiO_2_, Si, and MgAl_2_O_4_. As a rough estimation in a recent report [1], more than a million tons of the SAD are produced throughout the world each year. Nevertheless, it should be noted that nearly 95% of the SAD is dumped in landfills, which imposes a severe menace to the ecosystem [2]. For example, several substances such as F^−^, Cl^−^, and other toxic metal ions might leak into ground water consequently resulting in serious water pollution [3]. Inhalation of the SAD particles suspended in the air may cause some respiratory diseases [4]. In addition, when the AlN in the SAD comes in touch with water, water vapor, or moisture, hazardous ammonia gas (NH_3_) is released, which pollutes the atmosphere [5].

As mentioned above, dumping of the SAD will intensify environmental and health problems. At the same time, relatively higher amount of valuable aluminum element, which accounts for approximately 40–50 wt.% in the SAD [6,7], will be lost. In order to address these issues, the adoption of an appropriate recycling strategy for the SAD is of particular importance, and recently the synthesis of high value-added products (such as hydrotalcites, MgAl_2_O_4_ spinel, γ-alumina, α-alumina, zeolites, calcium aluminates, etc.,) from the SAD as a source of aluminum has attracted significant attention of researchers [8,9,10,11,12,13]. Cryolite (Na_3_AlF_6_) is an important industrial chemical which is extensively applied in industries, especially in the electrolytic process for aluminum production [14]. As for conventional synthesis of cryolite, relatively expensive industrial Al(OH)_3_ or Al_2_(SO_4_)_3_ tend to be chosen as the aluminum source, which increases the production cost of cryolite, and thus their practical use is limited to some extent. To our knowledge, the investigation on the use of cheap and readily available SAD as a potential resource for cryolite synthesis has not yet been reported.

Currently, hydrometallurgical process is a common technique to manage the SAD. The extraction of aluminum from the SAD is achieved by leaching operations. Aluminum can be leached out of the SAD using acidic or alkaline media, making the media concentrated in Al^3+^ or (Al(OH)_4_)^−^ ions [6,9,15,16]. Aluminum leached into the solution can be used to synthesize valuable products. Numerous literatures have indicated that the extraction efficiency of Al from the aluminum dross in acidic leaching was 20–30% higher than that in alkaline leaching [17,18,19,20]. However, it is worth noting that higher purity of Al in the alkaline leachate can be achieved compared to acidic leaching, because of the fact that most of the base metals (Cu, Fe, Ca, Mg, etc.,) in the aluminum dross are insoluble in the alkaline media but soluble in the acidic media [15]. The presence of a large number of metal ions are not desirable for cyolite preparation, during which the contents of impurities are limited strictly. In view of the above, under the premise of further improving the extraction efficiency of Al, it is more suitable to use alkaline leaching route in the present work. In the studies by Guo et al. [21,22], a pyro-hydrometallurgical process, which referred to low-temperature (below 900 °C) alkaline smelting and subsequent water leaching under atmospheric pressure, was found to be a feasible method to realize a high efficient extraction of Al from the SAD, and the corresponding extraction efficiency of Al was as high as 87.52%, which was about 30% higher than that in conventional alkaline leaching [20] and, in other words, equivalent to that in conventional acidic leaching [17,18,19]. Nevertheless, interestingly, a higher amount of Si can be introduced into the alkaline leachate from aluminum dross as a result of the dissolution of amphoteric metal (Si) by the NaOH [15]. Kobayashi et al. [23] mentioned that the presence of Si impurity in cryolite used widely in electrolytic process can cause significant lowering of the current efficiency. Therefore, for the sake of obtaining pure leachate as a preparation for subsequent cryolite synthesis, the removal of Si impurity from the leachate was indispensable.

In this study, a novel eco-friendly three-step process to synthesize cryolite from the SAD was developed. It consisted of water-washing pretreatment of SAD, extraction of Al component via pyro-hydrometallurgy (including low-temperature alkaline smelting, water leaching and Si removal from the leachate in sequence), and precipitation of cryolite by carbonation method. In order to realize comprehensive utilization of SAD efficiently, the influences of various processing parameters for each procedure were carefully investigated. In addition, as for the cryolite synthesized under optimal processing conditions, its chemical composition, ignition loss, phase, and morphology were characterized by reliable techniques.

## 2. Experimental

### 2.1. Materials

Secondary aluminum dross (SAD, −100 + 150 mesh (referred to those powders which can pass through the 100-mesh sieve rather than the 150-mesh sieve)) was derived from a domestic secondary aluminum plant. NaOH, NaNO_3_, CaO, Na_2_SiF_6_, and Na_2_CO_3_ were analytical grade and purchased from Aladdin Chemistry Co. Ltd. (Shanghai, China). Carbon dioxide gas (CO_2_) with a purity of 99.99% was obtained from Dongguan Yuanbang Gas Co. Ltd. (Dongguan, China). Deionized water was utilized at all stages of the experiments.

### 2.2. Synthesis Processes of Cryolite

**Figure 1:**
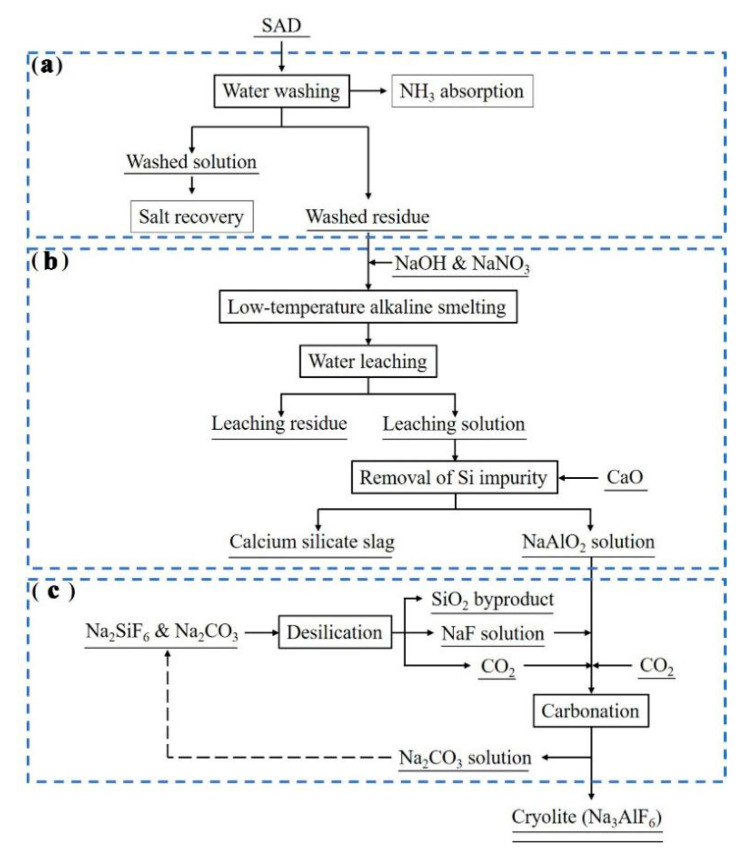
Flowchart for the synthesis of cryolite from secondary aluminum dross (SAD): water-washing pretreatment of SAD (**a**), extraction of Al component via pyro-hydrometallurgical process (**b**), and carbonation (**c**).

The procedure of cryolite synthesis in the current study contains three different steps as depicted in Figure 1: water-washing pretreatment of SAD (a); extraction of Al component via pyro-hydrometallurgical process (b); carbonation (c).

#### 2.2.1. Water-Washing Pretreatment of SAD 

Water-washing pretreatment of SAD (see Figure 1a) was carried out in a breaker flask which was placed on a constant temperature magnetic stirrer used for heating and stirring. First, a certain amount of SAD was loaded into the glass reactor. Then a given amount of deionized water was added into it. The main chemical reaction involved in the water-washing of SAD was as follows [5]:

(R1) $$\mathrm{AlN}+3H_{2}O=\mathrm{Al}{\left(\mathrm{OH}\right)}_{3}+{\mathrm{NH}}_{3}↑$$

The hydrolyzing of AlN in the SAD began at a given temperature with a stirring speed of 300 rpm, and the NH_3_ generated from the reactor was absorbed in the H_2_SO_4_ solution. After a predetermined reaction time, the content of the reactor was filtered under vacuum. The resulting washed solution was evaporated to obtain soluble salts while the remaining washed residue dried at 120 °C for 4 h and weighted. The following Equation (1) was used to calculate the hydrolysis efficiency of AlN:

(1) $$E_{1}=\frac{{\omega (\mathrm{AlN})}_{1}W_{1}{-\omega (\mathrm{AlN})}_{2}W_{2}}{{\omega (\mathrm{AlN})}_{1}W_{1}}\times 100\%$$

where E_1_ is the hydrolysis efficiency of AlN, W_1_ and W_2_ are the total mass of the SAD before and after water washing experiment, respectively, and ω(AlN)_1_ and ω(AlN)_2_ are the mass fraction of AlN in the SAD before and after water washing experiment, respectively.

#### 2.2.2. Extraction of Al Component via Pyro-Hydrometallurgical Process 

As illustrated in the Figure 1b, at the beginning, aluminous constituents in the washed residue (washed dross) obtained in Section 2.2.1 were converted into the soluble salts by low-temperature alkaline smelting process. A muffle furnace was used for smelting operation. The smelting was conducted by mixing the washed dross (10 g) with NaOH and NaNO_3_ in specific proportions at a given temperature for a certain time. The NaOH and NaNO_3_ acted as the absorbent and oxidant, respectively. During the smelting, different reactions related to aluminous constituents and main impurities (such as Si and SiO_2_ particles) in the washed dross were taking place as follows:

(R2) $$\mathrm{Al}{\left(\mathrm{OH}\right)}_{3}+\mathrm{NaOH}={\mathrm{NaAlO}}_{2}+2H_{2}O$$

(R3) $${\mathrm{Al}}_{2}O_{3}+2\mathrm{NaOH}=2{\mathrm{NaAlO}}_{2}+H_{2}O$$

(R4) $$10\mathrm{Al}+4\mathrm{NaOH}+6{\mathrm{NaNO}}_{3}=10{\mathrm{NaAlO}}_{2}+3N_{2}↑+2H_{2}O$$

(R5) $$5\mathrm{AlN}+2\mathrm{NaOH}+3{\mathrm{NaNO}}_{3}=5{\mathrm{NaAlO}}_{2}+4N_{2}↑+H_{2}O$$

(R6) $$\mathrm{Si}+{\mathrm{SiO}}_{2}+4\mathrm{NaOH}+O_{2}=2{\mathrm{Na}}_{2}{\mathrm{SiO}}_{3}+2H_{2}O$$

Subsequently, the smelting products were subjected to leaching with deionized water at a given temperature. After a certain leaching time, the resulting solution was separated from solid residue by vacuum filtration, and then analyzed for the recovery percent of Al and Si components. Extraction efficiency of Al and Si from washed dross could be calculated according to the following Equation (2):

(2) $$E_{2}=\frac{\mathrm{cV}}{m\omega }\times 100\%$$

where E_2_ is the extraction efficiency of each element, c is the mass concentration of each element in the leaching solution, V is the volume of solution, m is the mass of washed dross, and ω is the mass fraction of each element in the washed dross.

For the removal of Si impurity from the leaching solution, a certain amount of CaO was added into 100 mL of leachate in a 200-mL glass beaker. The experiment was carried out at 90 °C with a stirring speed of 300 rpm for 60 min. The Si element in the leachate, existing as Na_2_SiO_3_, could react with Ca(OH)_2_ and NaAlO_2_ to generate calcium silicate slags [24,25], which were then separated from the solution by vacuum filtration. Eventually, the purified NaAlO_2_ solution was obtained.

#### 2.2.3. Carbonation

As shown in the Figure 1c, prior to the synthetic reaction of cryolite, fluorine source (NaF) was gained by the desilication process of low-cost Na_2_SiF_6_ as a by-product of the production of phosphatic fertilizer. The following desilication reaction occurred upon the mixing of the Na_2_SiF_6_ solution with Na_2_CO_3_ solution at 100 °C under proper agitation (500 rpm):

(R7) $${\mathrm{Na}}_{2}{\mathrm{SiF}}_{6}+2{\mathrm{Na}}_{2}{\mathrm{CO}}_{3}=6\mathrm{NaF}+{\mathrm{SiO}}_{2}+2{\mathrm{CO}}_{2}↑$$

After the reaction (Equation R7) completed, the slurry was filtered under vacuum to remove the suspended SiO_2_ particles, and then the supernatant (NaF solution) was collected. Afterwards, the as-prepared NaF solution used as a precursor for fluorine was added into the purified NaAlO_2_ solution obtained in Section 2.2.2. Meanwhile, the CO_2_ (99.99% purity) under a pressure of 0.2 MPa was injected into the solution mixture with the gas flow rate of 0.5 L/min. The following carbonation reaction began at the temperatures ranged from 25 °C to 90 °C with a stirring speed of 300 rpm:

(R8) $$6\mathrm{NaF}+{\mathrm{NaAlO}}_{2}+2{\mathrm{CO}}_{2}={\mathrm{Na}}_{3}{\mathrm{AlF}}_{6}+2{\mathrm{Na}}_{2}{\mathrm{CO}}_{3}$$

Finally, the precipitated cryolite was separated from Na_2_CO_3_ solution by vacuum filtration, washed and dried at 120 °C for 4 h. Interestingly, as seen from the Equations R7 and R8, the Na_2_CO_3_ and CO_2_ can be recovered for recycling.

### 2.3. Methods of Characterization

The crystalline phases of the samples were identified by X-ray poly-crystalline diffractometer (XRD, Bruker D8 Advance) with Cu kα (*λ* = 1.5418 Å) radiation at an operating voltage of 40 kV with current of 40 mA. The chemical compositions of the SAD and as-synthesized cryolite were examined by X-ray fluorescence spectrometer (XRF, Axios Pw4400, Axios, Alemlo, The Netherlands), respectively. The Al and Si elements extracted in the leaching solution were detected by inductively coupled plasma atomic emission spectrometer (ICP-AES, JY ULTIMA-2, HORIBA JY, Paris, France). The variation in content of AlN in the SAD before and after the water-washing was measured by the Kjeldahl method. The contents of Na_2_O and Al_2_O_3_ in the leaching solution were analyzed by chemical titration method. The content of SiO_2_ in the leaching solution was determined according to the molybdenum blue photometric method (YS/T 575.3-2007 [26]). The evaluation of ignition loss of as-synthesized cryolite was carried out based on the YS/T 273.2-2006 standard [27]. After both the as-synthesized cryolite and the commercial cryolite were coated with a thin gold layer, their morphologies were observed using a scanning electron microscope (SEM, ZEISS Sigma 500, ZEISS, Jena, Germany) at an operating voltage of 3 kV. Besides, this microscope was equipped with an energy dispersive X-ray spectrometer (EDX, Oxford Instrument, Oxford, UK) for elemental analysis of samples.

## 3. Results and Discussion

### 3.1. Characterization of As-Received SAD

**Figure 2:**
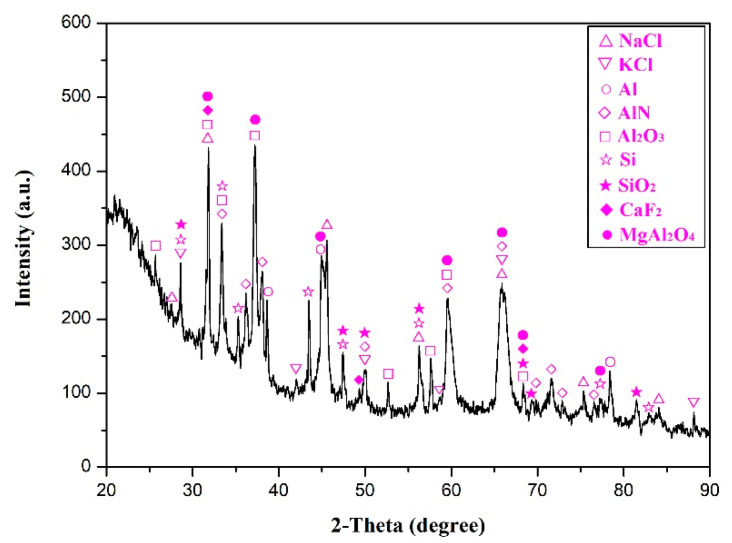
The XRD pattern of the as-received SAD.

Table 1 presents the chemical composition of the as-received SAD. It can be seen that, nearly 75 wt.% of the as-received SAD consisted of Al and O elements, and the rest of them were composed of salts constituents like Na, K, F, and Cl, and other alloying elements (Mg, Si, Ca, Fe, Ti, Mn, Cu, etc.,). In addition, the as-received SAD also contained some trace elements with content below 0.10 wt.% such as S, Cr, Sr, Zn, and Hg. The corresponding XRD pattern of the as-received SAD is depicted in Figure 2. According to the diffraction peaks, it can be confirmed that the as-received SAD was mainly constituted of nine crystalline phases: sodium chloride (NaCl), potassium chloride (KCl), pure aluminum (Al), aluminum nitride (AlN), corundum (Al_2_O_3_), silicon (Si), silica (SiO_2_), calcium fluoride (CaF_2_), and spinel (MgAl_2_O_4_). It is worth mentioning that certain other phases containing the elements as shown in Table 1 were not detected by XRD, which is attributed to the fact that they may be in an amorphous state or exist as crystalline substances but in very low amounts [8,28].

### 3.2. Water-Washing Pretreatment of SAD

Prior to the Al extraction, it is essential for the SAD to be subjected to the water-washing pretreatment. On the one hand, the amount of AlN constituent possessing high reactivity with water can be decreased remarkably, thereby enhancing the chemical stability of the SAD as a raw material for cyolite production. Meanwhile, the NH_3_ generated from the hydrolysis of AlN can be absorbed by acid to generate ammonium salt fertilizer, thus eliminating the hazard of NH_3_ and simultaneously achieving the recovery of nitrogen in the SAD. On the other hand, water-soluble salts constituents like NaCl and KCl in the SAD can be recovered back by washing and evaporation. More importantly, the hydrolysis of AlN and the dissolution of salts contribute to increasing the efficiency of the subsequent leaching process [16,17].

#### 3.2.1. Hydrolysis of AlN in the SAD

Regarding the SAD, in order to maximize the removal of the AlN constituent and simultaneously realize the centralized recovery of nitrogen as much as possible, one-factor-at-a time (OFAT) method was adopted to examine the effects of water-washing processing parameters such as temperature, reaction time and liquid-to-solid ratio on the hydrolysis efficiency of AlN in the SAD calculated by Equation (1), eventually optimizing the water-washing process. As for the OFAT method, single factor experiments are repeated until an optimal combination of factors is found. Here, the ranges of influencing factors were set as follows: temperature (25–90 °C), reaction time (2–6 h), and liquid-to-solid ratio (5–13 mL/g). In each experiment, the dosage of SAD was fixed at 10 g.

**Figure 3:**
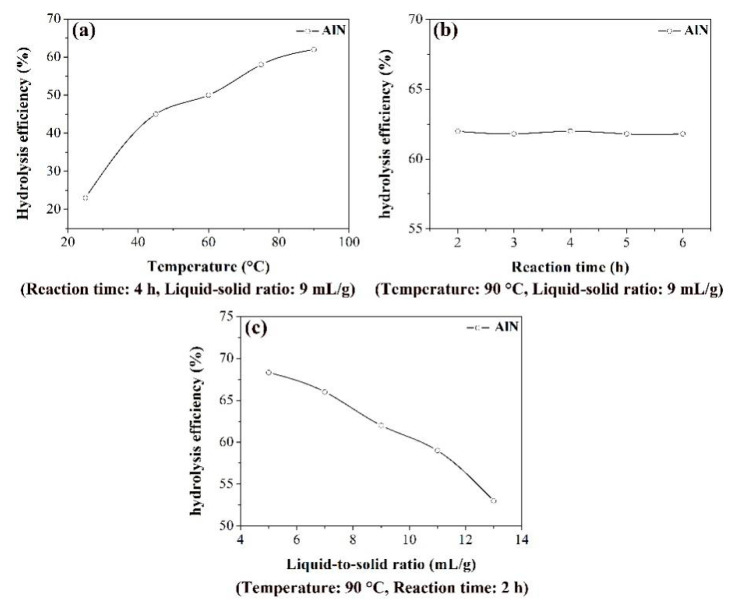
Effects of temperature (**a**), reaction time (**b**), and liquid-to-solid ratio (**c**) on the hydrolysis efficiency of AlN.

Figure 3 illustrates the effects of the water-washing conditions on the hydrolysis efficiency of AlN. It can be observed from the Figure 3a that when the temperature varied from 25 °C to 90 °C, the hydrolysis efficiency of AlN was increased continuously. A higher temperature can promote the dissolution of salts on the surface of the AlN particles and make the water penetrate quickly into the AlN [5], thereby ensuring the proceeding of the hydrolysis process more adequately. Besides, with the increase in the temperature, the precipitation of NH_3_ occurred more rapidly because of the decreasing of the solubility of NH_3_, thus promoting the proceeding of forward reaction of hydrolysis, i.e., AlN + 3H_2_O → Al(OH)_3_ + NH_3_↑. Therefore, 90 °C was selected as the optimal water-washing temperature. Figure 3b shows the relationship between reaction time and hydrolysis efficiency of AlN. As the reaction time was extended from 2 h to 6 h, the hydrolysis efficiency of AlN remained almost constant, indicating the completion of hydrolysis reaction within 2 h. Hence, 2 h was chosen as the optimal water-washing time. By taking the temperature of 90 °C and the reaction time of 2 h for the water-washing process, a variation of liquid-to-solid ratio on the hydrolysis efficiency of AlN was investigated to determine the optimal liquid-to-solid ratio, and the corresponding results are described in Figure 3c. Apparently, the hydrolysis efficiency of AlN exhibited a decreasing trend with increasing the liquid-to-solid ratio. According to the study of Li et al. [5], they found that the hydrolysis of AlN was an exothermic reaction using thermodynamic calculation. Based on this fact, a higher temperature of the reaction system appears at a lower liquid-to-solid ratio, thereby resulting in the adequate hydrolysis of AlN in the SAD. Consequently, the liquid-to-solid ratio of 5 mL/g was taken as the optimal value for the water-washing process.

In summary, the optimal water-washing conditions were obtained as follows: temperature of 90 °C, reaction time of 2 h, and liquid-to-solid ratio of 5 mL/g. Under these conditions, the hydrolysis efficiency of AlN could reach 68.3%, and through a rough calculation, it was found that approximately 38 kg of NH_3_ can be produced after the water-washing pretreatment of one metric ton of the SAD used in this study.

#### 3.2.2. Recovery of Salts in the SAD

**Figure 4:**
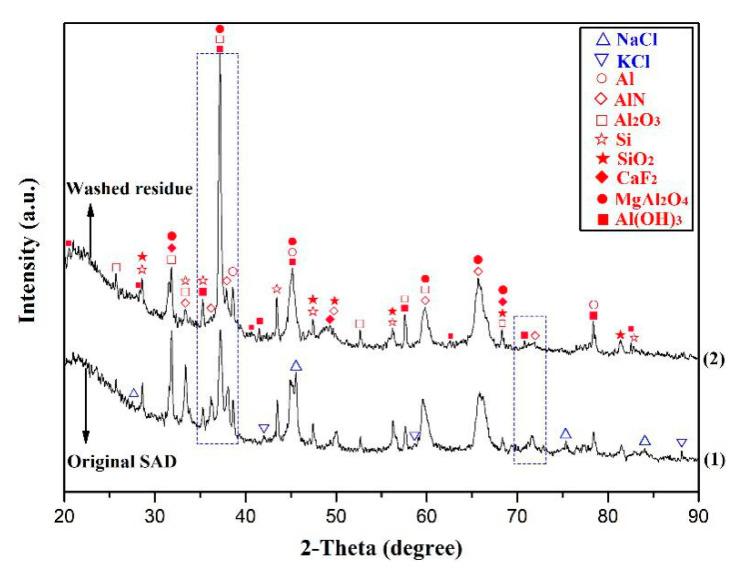
The XRD patterns of the SAD before and after water washing: (1) original SAD, (2) washed residue.

Under the optimal water-washing conditions mentioned above, 100 g of SAD was subjected to the washing process. Figure 4 shows the XRD patterns of the SAD before and after water washing. As compared to the original SAD, the peaks of Al, AlN, Al_2_O_3_, Si, SiO_2_, CaF_2_, and MgAl_2_O_4_ remained in the XRD pattern of washed residue while the peaks of soluble salts like NaCl and KCl disappeared. Thus, it may be concluded that both NaCl and KCl have been effectively removed by the water-washing of the SAD. In addition, another major phase of Al(OH)_3_ in the washed residue was also detected, which was derived from the hydrolysis process of AlN in the SAD. As seen from the areas marked by blue dotted squares in Figure 4, the peak intensity of the AlN was remarkably weakened after the water washing of the SAD, due to the hydrolyzing depletion of AlN. As for the obtained washed solution, it was evaporated to get the mixed NaCl and KCl crystals. As expected, it would be economically beneficial to reuse the recovered salts in the secondary aluminum smelter.

### 3.3. Extraction of Al Component via Pyro-Hydrometallurgical Process

#### 3.3.1. Effect of Different Factors on the Extraction Efficiency of Al

As mentioned in Section 2.2.2, the Al component in the washed dross can be extracted via pyro-hydrometallurgical process. Meanwhile, the main impurity element (Si) will also be introduced into the leachate. Under the premise of the obtained optimal water-washing process, in order to get maximum extraction rate of Al and simultaneously suppress the leaching of Si efficiently, the OFAT approach was employed to optimize the pyro-hydrometallurgical process. The ranges of different factors influencing the extraction efficiency of Al and Si calculated by Equation (2) were set as follows: alkali (NaOH)-to-dross mass ratio (0.9–1.7), salt (NaNO_3_)-to-dross mass ratio (0.1–0.9), smelting time (40–120 min), leaching temperature (50–90 °C), and leaching time (20–100 min). In each experiment, the smelting temperature was specified to be 500 °C, which was found to be the optimal temperature to extract Al from aluminum dross in the low-temperature alkaline smelting on the basis of the study by Guo et al. [21]. Besides, for the sake of making the smelting products fully dissolve in water during leaching, the ratio of deionized water to smelting products was fixed at 6 mL/g.

- Effect of alkali-to-dross mass ratio

**Figure 5:**
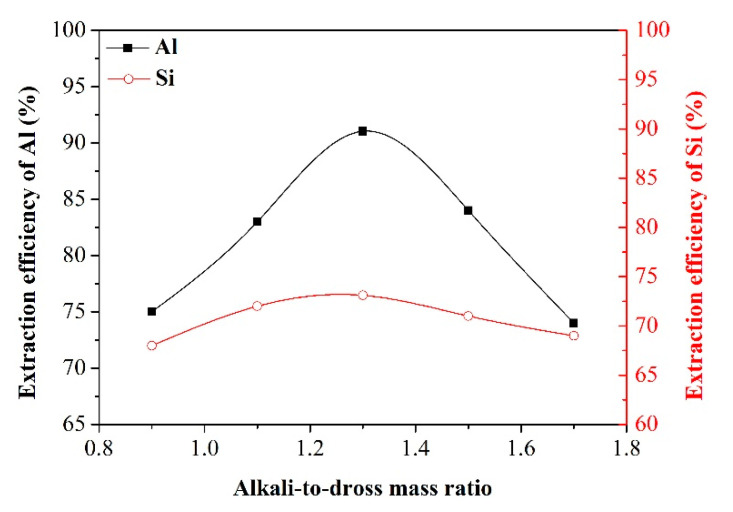
Effect of alkali-to-dross mass ratio on the extraction efficiency of Al and Si.

To evaluate the effect of alkali-to-dross mass ratio on the extraction efficiency of Al and Si, salt-to-dross mass ratio, smelting time, leaching temperature, and leaching time were considered at specified values of 0.5, 80 min, 70 °C, and 60 min, respectively. Figure 5 shows the variation of extraction efficiency versus alkali-to-dross mass ratio. As the alkali-to-dross mass ratio was increased from 0.9 to 1.3, the extraction efficiency of Al and Si was increased accordingly. This may be ascribed to the fact that the increasing of OH^–^ ion activity in the reaction system is beneficial for the formation of NaAlO_2_ and Na_2_SiO_3_ (see Equations (R2)–(R6)). However, further increase of alkali-to-dross mass ratio resulted in a decrease in leaching efficiency of Al and Si. This can be due to the fact that excessive dosage of NaOH increases the viscosity of reaction system and thus decreases the rate of mass transfer [21]. Therefore, considering the principle of achieving a higher Al concentration and lower Si concentration simultaneously in the leachate, the alkali-to-dross mass ratio of 1.3 was selected as the optimal value for the extraction process and the subsequent single factor experiments will be conducted at this alkali-to-dross mass ratio.

- Effect of salt-to-dross mass ratio

**Figure 6:**
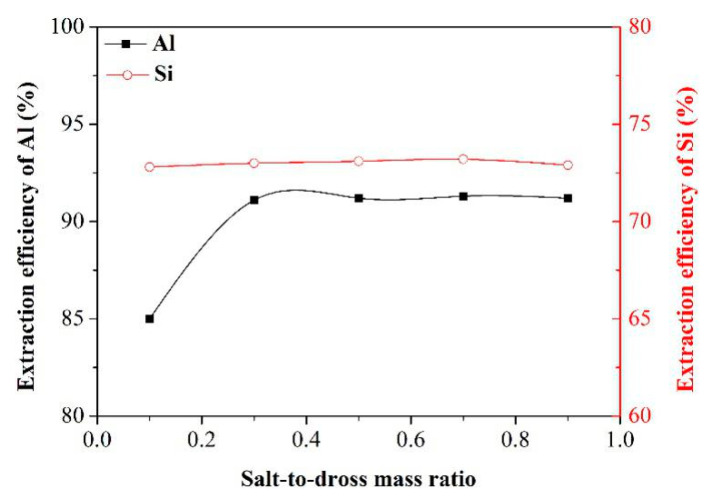
Effect of salt-to-dross mass ratio on the extraction efficiency of Al and Si.

The investigation on the effect of salt-to-dross mass ratio was performed under the following experimental conditions: alkali-to-dross mass ration of 1.3, smelting time of 80 min, leaching temperature of 70 °C, and leaching time of 60 min. Figure 6 presents the effect of salt-to-dross mass ratio on the extraction efficiency of Al and Si. Apparently, with the salt-to-dross mass ratio increasing, the extraction efficiency of Si was nearly constant, whereas the extraction efficiency of Al was increased initially and thereafter kept stable. As for the Si extraction, the formation of water-soluble Na_2_SiO_3_, represented by Equation (R6), cannot be affected by the addition of NaNO_3_. During the progress of Al extraction, the NaNO_3_, acted as a strong oxidizer, can promote the oxidation of Al and AlN constituents in the washed dross (see Equations (R4) and (R5)), and then more oxidation products (Al_2_O_3_) might react with alkali to form the water-soluble NaAlO_2_ (see Equation (R3)). When the salt-to-dross mass ratio reached 0.3, reactants of Al and AlN were exhausted. According to the above-mentioned analysis, the salt-to-dross mass ratio of 0.3 was considered as the optimal value for the extraction process and the influence of other factors on the extraction efficiency of Al and Si will be studied at this salt-to-dross mass ratio.

- Effect of smelting time

**Figure 7:**
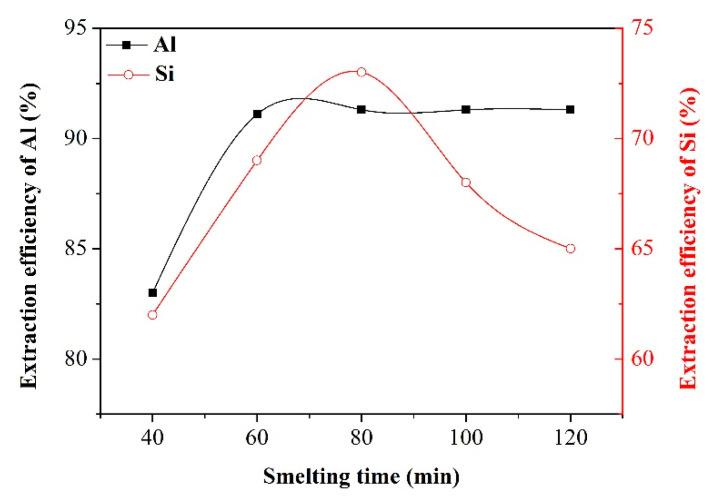
Effect of smelting time on the extraction efficiency of Al and Si.

The following experimental conditions were applied to investigate the effect of smelting time on the extraction efficiency of Al and Si: alkali-to-dross mass ratio of 1.3, salt-to-dross mass ratio of 0.3, leaching temperature of 70 °C, and leaching time of 60 min. The results are illustrated in the Figure 7. As the smelting time was extended from 40 min to 60 min, the extraction efficiency of Al was increased accordingly. At a shorter smelting time, inadequate leaching reactions led to a lower extraction rate of Al. Nevertheless, when the smelting time was longer than 60 min, the extraction efficiency of Al remained at a nearly constant value, suggesting the completion of leaching reactions. As for the Si extraction, when the smelting time was shorter than 80 min, the leaching efficiency of Si was increased with the proceeding of leaching reaction. Moreover, increasing smelting time beyond 80 min had an adverse effect on the Si extraction. This observation is explained by the possibility that SiO_2_ can be combined with certain substances in the reaction system to generate water-insoluble composite oxide particles (such as 3Al_2_O_3_·2SiO_2_ and 2CaO·SiO_2_) under longer time of smelting. Consequently, the smelting time of 120 min, as an optimal value for the extraction process, was chosen to study other factors affecting the extraction efficiency of Al and Si.

- Effect of leaching temperature

**Figure 8:**
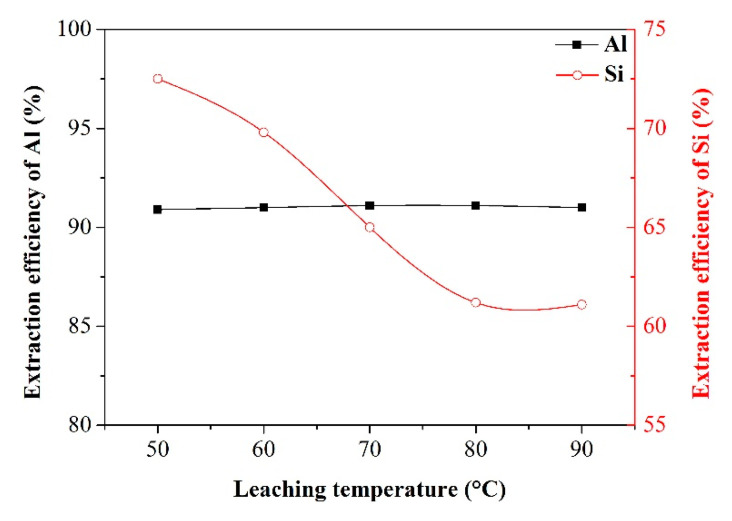
Effect of leaching temperature on the extraction efficiency of Al and Si.

After the smelting operation, as-produced NaAlO_2_ and Na_2_SiO_3_ were dissolved in water by water leaching, realizing the separation of Al and Si with other impurities. To investigate the effect of leaching temperature on the extraction efficiency of Al and Si, the following experimental conditions were considered: alkali-to-dross mass ratio of 1.3, salt-to-dross mass ratio of 0.3, smelting time of 120 min, and leaching time of 60 min. Figure 8 shows the effect of leaching temperature on the extraction efficiency of Al and Si. It is apparent that, with increasing the leaching temperature, the extraction efficiency of Al remained almost the same level, whereas the extraction efficiency of Si was decreased first and then kept at a stable trend. The decline in the extraction efficiency of Si may be due to the fact that the elevated temperature of solution system promotes silicon suboxide to react with other substances to produce precipitates readily separated from the leachate. According to the results shown in Figure 8, the leaching temperature of 80 °C was chosen for the subsequent extraction efficiency experiments.

- Effect of leaching time

**Figure 9:**
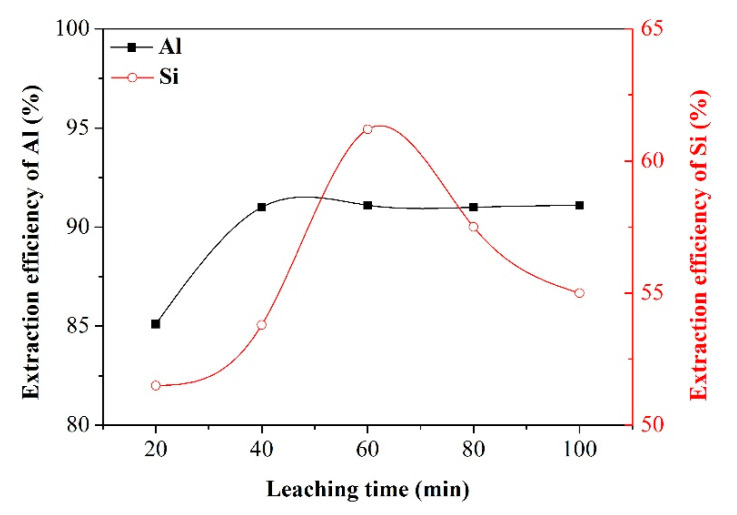
Effect of leaching time on the extraction efficiency of Al and Si.

In view of the water leaching, besides the leaching temperature, an appropriate leaching time also plays a significant role in the extraction of Al and Si. The research on the effect of leaching time on the extraction of Al and Si was conducted under the following optimal conditions based on previous experimental results: alkali-to-dross mass ratio of 1.3, salt-to-dross mass ratio of 0.3, smelting time of 120 min, and leaching temperature of 80 °C. Figure 9 illustrates the variation of extraction efficiency versus leaching time. Under the condition of leaching time varied from 20 min to 40 min, the extraction efficiency of Al and Si exhibited an increasing trend, due to the proceeding of dissolution of NaAlO_2_ and Na_2_SiO_3_. With further increasing the leaching time beyond 40 min, the extraction efficiency of Al remained almost constant while the extraction efficiency of Si was increased continuously. But it should be noticed that the extraction efficiency of Si began to decrease when the leaching time exceeded 60 min. This phenomenon might be ascribed to the possibility that SiO_2_ reacts with Na_2_O and Al_2_O_3_ to form sodium-silicon residue in the solution system. As a result, the leaching time of 40 min was taken as the optimal value for the extraction process.

In summary, the optimal conditions of pyro-hydrometallurgical process were obtained as follows: alkali-to-dross mass ratio of 1.3, salt-to-dross mass ratio of 0.3, smelting temperature of 500 °C, smelting time of 120 min, deionized water to smelting products ratio of 6 mL/g, leaching temperature of 80 °C, and leaching time of 40 min. Under these conditions, the extraction efficiency of Al reached up to 91.5%, which was 34% higher than that obtained in conventional alkaline leaching [20]. At the same time, the extraction efficiency of Si was limited to 53.8%.

#### 3.3.2. The Removal of Si Impurity from NaAlO_2_ Solution

Under optimal conditions of pyro-hydrometallurgical process, the resulting leaching solution was obtained. To acquire the pure NaAlO_2_ solution, the deep desilication approach by adding CaO [29] was implemented to effectively remove Si impurity from the leachate. The corresponding reaction was taken place as below [30]:

(R9) $$3\mathrm{Ca}{\left(\mathrm{OH}\right)}_{2}+{\mathrm{Na}}_{2}{\mathrm{SiO}}_{3}+2\mathrm{NaAl}{\left(\mathrm{OH}\right)}_{4}=3\mathrm{CaO}\cdot {\mathrm{Al}}_{2}O_{3}\cdot {\mathrm{SiO}}_{2}\cdot 4H_{2}O+4\mathrm{NaOH}+H_{2}O$$

After the completion of the reaction (Equation (R9)), the calcium silicate slag, i.e., 3CaO·Al_2_O_3_·SiO_2_·4H_2_O, was separated from the NaAlO_2_ solution, thereby realizing the removal of Si impurity. Table 2 shows the mass concentrations of major components of NaAlO_2_ solution before and after the purification. As can be seen, the silica module, i.e., the mass ratio of Al_2_O_3_ to SiO_2_, was increased from 20 to 449, and the desilication rate reached up to nearly 96%. These results indicated a better purifying effect of NaAlO_2_ solution by CaO addition, which will be very beneficial for the subsequent synthesis of high-quality cryolite.

### 3.4. Synthesis of Cryolite (Na_3_AlF_6_) by Carbonation Method

#### 3.4.1. Effect of Different Factors on the Quality of Synthetic Cryolite

The Chinese national standard of GB/T 4291-2017 has already put forward stringent requirements on the quality of synthetic cryolite, mainly involving the chemical composition and ignition loss of the cryolite [31]. Thereinto, the level of crystal water and volatile impurity phases in the cryolite can be denoted by the ignition loss. Apparently, the higher the ignition loss, the lower the utilization of cryolite. In general, during the reaction process of carbonation represented by Equation (R8), in addition to the principal phase of Na_3_AlF_6_, the cryolite products can also contain several impurity phases that will directly influence the purity of main product. Under the premise of the obtained optimal water-washing and Al extraction processes, in order to effectively search for the optimal conditions of carbonation process to precipitate high-quality synthetic cryolite, a comprehensive study was carried out to reveal the effects of reaction temperature, NaAlO_2_ concentration and F/(6Al) molar ratio on the aluminum content, fluorine content, and ignition loss of synthetic cryolite. To optimize the quality of synthetic cryolite, the OFAT method was adopted. The ranges of above-mentioned factors were set as follows: reaction temperature (30–90 °C), NaAlO_2_ concentration (0.02–0.14 mol/L), and F/(6Al) molar ratio (0.90–1.10). In each experiment, the pH value of reaction system was adjusted to 9.5 which has been confirmed to be the optimal pH value to synthesize cryolite in our preliminary experiments of the present study.

- Effect of reaction temperature

**Figure 10:**
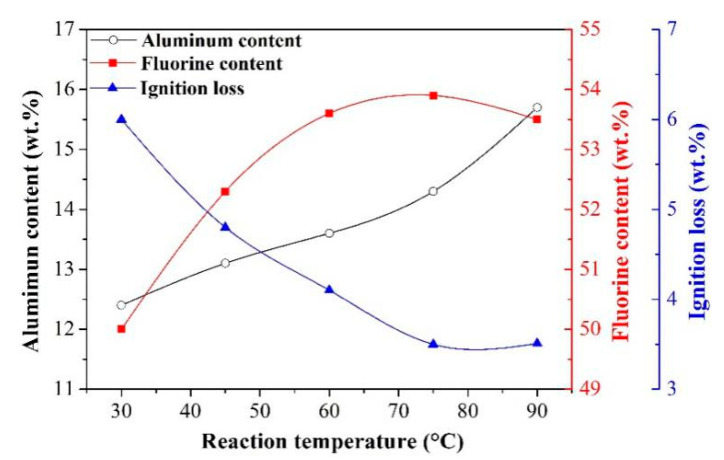
Effect of reaction temperature on the aluminum content, fluorine content, and ignition loss of synthetic cryolite.

The investigation on the effect of reaction temperature was carried out under the following experimental conditions: NaAlO_2_ concentration of 0.08 mol/L, F/(6Al) molar ratio of 1.00. Figure 10 presents the effect of reaction temperature on the aluminum content, fluorine content, and ignition loss of synthetic cryolite.

According to Figure 10, as the temperature was increased from 30 °C to 75 °C, the fluorine content in the cryolite was increased while the ignition loss of cryolite was decreased. However, when the temperature exceeded 75 °C, the fluorine content in the cryolite began to decrease while the ignition loss of cryolite remained almost the same level. Regarding the aluminum content of cryolite, it was increased continuously when the temperature varied within the range from 30 °C to 90 °C. These evolution trends can be attributed to the following two reasons. On the one hand, the dawsonite (NaAlCO_3_(OH)_2_·2H_2_O) will be formed during the carbonation process according to the reaction proposed by Bénézeth et al. [32]:

(R10) $${\mathrm{NaAlO}}_{2}+{\mathrm{CO}}_{2}+3H_{2}O={\mathrm{NaAlCO}}_{3}{\left(\mathrm{OH}\right)}_{2}\cdot 2H_{2}O$$

In addition, they also found that the solubility of dawsonite was increased notably with increasing temperature [32]. Namely, the higher the reaction temperature, the less the amount of dawsonite precipitates. With the change in temperature lying in the range from 30 °C to 75 °C, the relative amount of fluorine-free dawsonite was decreased, thus resulting in an increase of fluorine content in the cryolite products. Meanwhile, the reduction in the content of crystal waters relevant to the dawsonite would lead to a decline in the ignition loss of cryolite products. On the other hand, the following reaction is induced during the process of synthesizing cryolite under alkaline environment [33]:

(R11) $$3{\mathrm{OH}}^{-}+{\mathrm{Al}}^{3+}=\mathrm{Al}{\left(\mathrm{OH}\right)}_{3}$$

Through thermodynamic calculation, the formation of Al(OH)_3_ precipitate belonged to an endothermic reaction (Equation (R11)) [33]. Theoretically, the higher the reaction temperature, the more the amount of Al(OH)_3_ precipitates. Furthermore, it should be noted that the aluminum element in Al(OH)_3_ (34.6 wt.%) was significantly higher than that in Na_3_AlF_6_ (12.9 wt.%). Thus, the continuously rising trend of aluminum content in the cryolite products appeared with the temperature raising. Besides, owing to the increment in relative amount of Al(OH)_3_, the fluorine content in the cryolite products exhibited a declining trend when the temperature varied from 75 °C to 90 °C. Eventually, based on the above-mentioned results, 75 °C was selected as the optimal reaction temperature to investigate other factors affecting the quality of synthetic cryolite.

- Effect of NaAlO_2_ concentration

**Figure 11:**
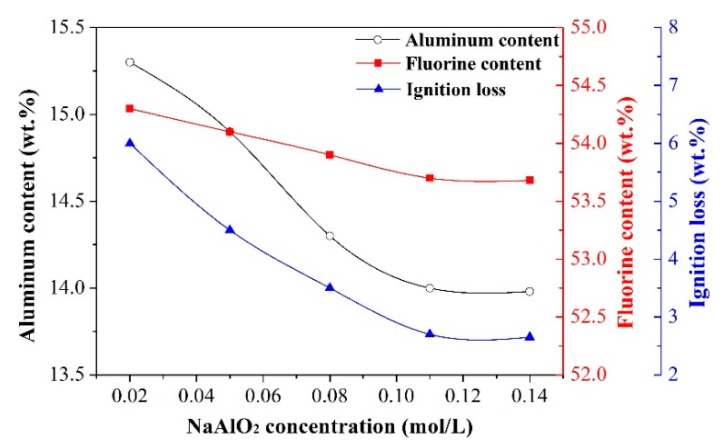
Effect of NaAlO_2_ concentration on the aluminum content, fluorine content, and ignition loss of synthetic cryolite.

The following experimental conditions were adopted to study the effect of NaAlO_2_ concentration on the aluminum content, fluorine content, and ignition loss of synthetic cryolite: reaction temperature of 75 °C, F/(6Al) molar ratio of 1.00. The results are depicted in Figure 11.

It can be clearly seen from Figure 11 that the aluminum content, fluorine content, and ignition loss of synthetic cryolite were decreased first and then kept unchanged with the NaAlO_2_ concentration increasing. It is worthy to mention that the falling trend of the aluminum content was more significant than that of the fluorine content. A possible explanation for this phenomenon is that, the increasing of Na^+^ ion concentration can promote the transformation of NaAlF_4_ into Na_3_AlF_6_ according to the reaction [34]:

(R12) $${\mathrm{Na}}_{3}{\mathrm{AlF}}_{6}={\mathrm{NaAlF}}_{4}+2{\mathrm{Na}}^{+}+2F^{-}$$

Herein, after the phase transformation of equimolar cryolite completed, it was calculated that the extent of decline of the aluminum content was almost four times greater than that of the fluorine content. Furthermore, Veryasov et al. [35] pointed out that the roles of NaAlF_4_ and Na_3_AlF_6_ were respectively equivalent to NaF·AlF_3_·0.83H_2_O and 3NaF·AlF_3_·0.167H_2_O in the system of NaF-AlF_3_-H_2_O. Therefore, when the NaAlF_4_ phase was transformed into Na_3_AlF_6_ phase, the decreasing of crystal water content would cause the ignition loss of cryolite products decreasing. Interestingly, further increase of NaAlO_2_ concentration beyond 0.11 mol/L had little impact on the chemical composition of cryolite products. According to the above-mentioned findings, the NaAlO_2_ concentration of 0.11 mol/L was chosen for the subsequent single factor experiments.

- Effect of F/(6Al) molar ratio

**Figure 12:**
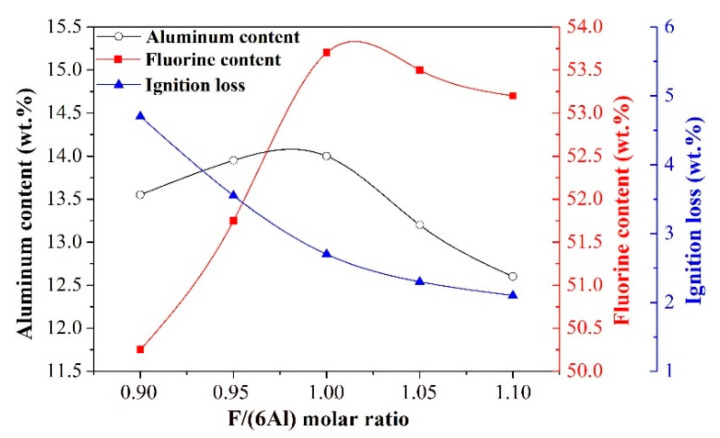
Effect of F/(6Al) molar ratio on the aluminum content, fluorine content and ignition loss of synthetic cryolite.

By taking the temperature of 75 °C and the NaAlO_2_ solution of 0.11 mol/L for the carbonation reaction, a variation of F/(6Al) molar ratio on the aluminum content, fluorine content, and ignition loss of synthetic cryolite was investigated to determine the optimal F/(6Al) molar ratio. The results are shown in Figure 12.

As can be seen from Figure 12, both the aluminum content and the fluorine content in the cryolite exhibited a rising trend when the F/(6Al) molar ratio varied from 0.90 to 1.00. Under a lower molar ratio of F/(6Al), the excessive NaAlO_2_ is likely to react with CO_2_ to form impurity phase (dawsonite). Moreover, the F^-^ ion with lower concentration could be combined with Al^3+^ ion to generate mainly AlF_4_^-^ ion. When the concentration of F^-^ ion was increased, the formation of fluorine-free dawsonite containing high crystal water was inhibited and thereby the generation of cryolite (NaAlF_4_) was promoted, resulting in a significant decline of ignition loss and remarkable enhancement in the fluorine content for cryolite products. Meanwhile, because of the aluminum content in the dawsonite (15 wt.%) being lower than that in the NaAlF_4_ (21.4 wt.%), the aluminum content in the cryolite products was increased to some extent. However, when the F/(6Al) molar ratio exceeded 1.00, all of the aluminum content, fluorine content, and ignition loss of synthetic cryolite were decreased continuously, as indicated in Figure 12. Under a higher molar ratio of F/(6Al), F^-^ ion with higher concentration seemed to be combined with Al^3+^ ion to generate mainly AlF_6_^3-^ ion. The formation of Na_3_AlF_6_ led to a diminution in the aluminum content, fluorine content, and ignition loss. As a result, the F/(6Al) molar ratio of 1.10 was taken as the optimal value for the carbonation reaction.

In summary, the optimal conditions of carbonation reaction (Equation (R8)) to produce high quality synthetic cryolite were obtained as follows: reaction temperature of 75 °C, NaAlO_2_ concentration of 0.11 mol/L, and F/(6Al) molar ratio of 1.10. Under these conditions, the aluminum content, fluorine content, and ignition loss of the synthetic cryolite could reach 12.6 wt.%, 53.2 wt.%, and 2.1 wt.%, respectively. In addition, further measurement by XRF was also performed, and its sodium and SiO_2_ impurity content were 32.18 wt.% and 0.125 wt.%, respectively.

#### 3.4.2. Characterization of As-Synthesized Cryolite

**Figure 13:**
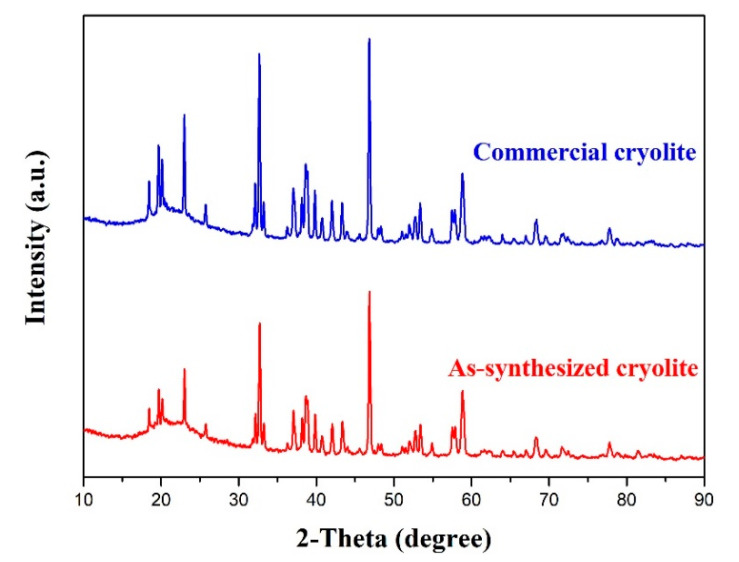
The XRD patterns of commercial cryolite and as-synthesized cryolite.

**Figure 14:**
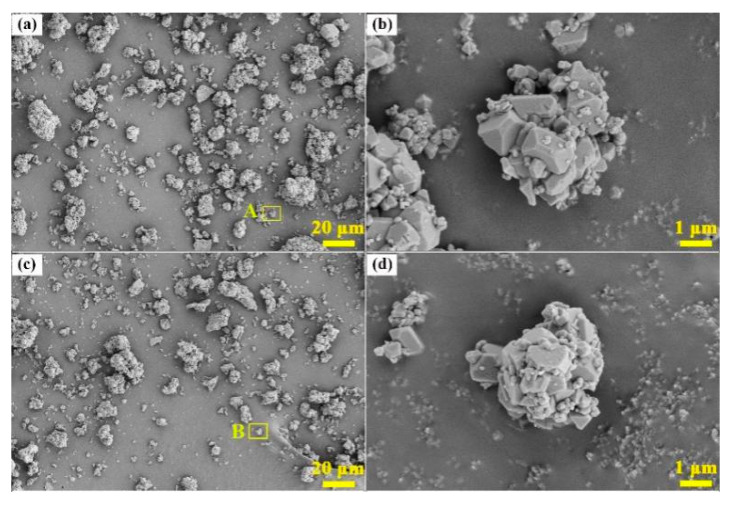
SEM micrographs of (**a**) commercial cryolite, (**b**) enlarged image of the area A marked in (**a**), (**c**) as-synthesized cryolite, and (**d**) enlarged image of the area B marked in (**c**).

**Figure 15:**
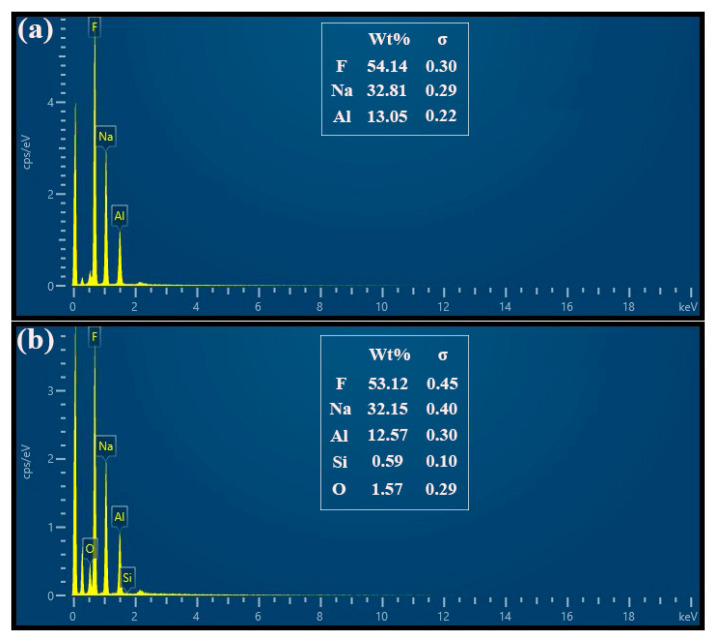
EDX analysis of (**a**) commercial cryolite and (**b**) as-synthesized cryolite.

Figure 13 illustrates the XRD patterns of commercial cryolite and as-synthesized cryolite. Apparently, the XRD characteristic peaks of as-synthesized cryolite matched very well with those of commercial cryolite, which confirmed that the main phase precipitated from the carbonation reaction under optimal conditions was Na_3_AlF_6_. Besides, it can also be observed that the characteristic peak intensity of the as-synthesized cryolite was relatively weaker than that of the commercial one. This may be ascribed to the fact that there exist a certain amount of impurities in the washed dross leachate influencing the purity of cryolite product. Figure 14 represents the SEM images of the commercial cryolite and as-synthesized cryolite. As seen from Figure 14a,c, the morphology of as-synthesized cryolite powders was similar with that of commercial cryolite powders. The powder consisted of several agglomerates of different sizes. Figure 14b,d revealed that the agglomerates were composed of fine particles (~1 μm size) in polyhedral shape which exhibited a strong tendency toward agglomeration. Figure 15 shows the EDX analysis of commercial cryolite and as-synthesized cryolite. The EDX results disclosed a major chemistry of Al, F, and Na as the main elements of cryolite. Interestingly, a trace amount of Si and O were detected as impurities in the as-synthesized cryolite rather than in the commercial cryolite. In addition, the contents of major components (Al, F, and Na) of as-synthesized cryolite as represented in Figure 15b were almost consistent with the XRF results as mentioned in Section 3.4.1. It was calculated that the molecule ratio of as-synthesized cryolite (defined as the molecule ratio of NaF/AlF_3_ or the molar ratio of Na/Al) was approximately three, which belonged to high molecular ratio.

As mentioned above, it is clear that the chemical composition and physical property of the as-synthesized cryolite with high molecular ratio (such as aluminum content, fluorine content, sodium content, SiO_2_ impurity content, and ignition loss) are well in line with the requirements of the Chinese national standard (GB/T 4291-2017 [31]). Besides, the obtained results of XRD and SEM/EDX analyses, as depicted in Figure 13, Figure 14, and Figure 15, indicated that the as-synthesized cryolite had nearly the same phase composition and morphological feature with the commercial one. Based on the above comprehensive analysis, we can finally conclude that the as-synthesized cryolite can be a promising candidate for industrial applications.

## 4. Conclusions

In this paper, a novel eco-friendly method to synthesize cryolite from the SAD was developed to realize comprehensive utilization of SAD efficiently. This method consisted of three different procedures: water-washing pretreatment of SAD, extraction of Al component via pyro-hydrometallurgy, and precipitation of cryolite by carbonation method. After systematic investigations, the following conclusions were drawn:

(1) The maximum hydrolysis efficiency of AlN in the SAD, i.e., 68.3%, was achieved when the water-washing temperature, reaction time, stirring speed, and liquid-to-solid ratio were 90 °C, 2 h, 300 rpm, and 5 mL/g, respectively. Meanwhile, approximately 38 kg of NH_3_ can be produced after the water-washing pretreatment of one metric ton of the SAD. In addition, the water-soluble salts constituents such as NaCl and KCl were recovered by washing and evaporation.
(2) The optimal conditions of pyro-hydrometallurgical process were obtained as follows: alkali (NaOH)-to-dross mass ratio of 1.3, salt (NaNO_3_)-to-dross mass ratio of 0.3, smelting temperature of 500 °C, smelting time of 120 min, deionized water to smelting products ratio of 6 mL/g, leaching temperature of 80 °C, and leaching time of 40 min. Under these conditions, the extraction efficiency of Al reached up to 91.5%, which was 34% higher than that achieved in conventional alkaline leaching.
(3) The synthetic cryolite of high quality was precipitated under the following optimal conditions of carbonation: synthesis temperature of 75 °C, NaAlO_2_ concentration of 0.11 mol/L, F/(6Al) molar ratio of 1.10, and 99.99% CO_2_ gas pressure and flow rate of 0.2 MPa and 0.5 L/min respectively. The XRD and SEM/EDX analyses suggested that, the as-synthesized cryolite mainly consisted of the Na_3_AlF_6_ phase, and despite the occurrence of agglomeration, its morphology still exhibited a polyhedral shape with ~1 μm size. Moreover, aluminum content (12.6 wt.%), fluorine content (53.2 wt.%), sodium content (32.18 wt.%), SiO_2_ impurity content (0.125 wt.%), and ignition loss (2.1 wt.%) of the as-synthesized cryolite with high molecular ratio complied well with the requirement of the Chinese national standard (GB/T 4291-2017). Besides, from the angle of aluminum source and fluorine source used to synthesize cryolite, this novel eco-friendly three-step process was of lower cost and higher efficiency compared to conventional synthetic methods, suggesting it had the potential to be applied to the cryolite industry.

## Figures and Tables

**Table 1:** Chemical composition of the as-received SAD (wt.%).

**Element**	**O**	**Al**	**Si**	**Na**	**Mg**	**F**	**Cl**	**K**	**Ca**
Content	31.68	42.87	3.54	5.71	1.68	2.06	7.56	2.39	1.22
**Element**	**Fe**	**Ti**	**Cu**	**Mn**	**S**	**Sr**	**Zn**	**Cr**	**Hg**
Content	0.19	0.41	0.35	0.17	0.09	0.02	0.02	0.03	0.01

**Table 2:** The mass concentrations of major components of NaAlO_2_ solution before and after the purification.

Stages	Major Components (g/L)		
	Al_2_O_3_	Na_2_O	SiO_2_
Before the purification	48.16	73.84	2.41
After the purification	43.52	71.59	0.097
